# 3D-bioprinted urethral grafts: Revolutionizing urethral stricture treatment

**DOI:** 10.1080/20905998.2025.2504797

**Published:** 2025-05-13

**Authors:** Kirolos Eskandar

**Affiliations:** General Medicine, Diakonie Klinik Mosbach, Mosbach, Germany

**Keywords:** 3D-bioprinting, urethral stricture, tissue engineering, regenerative medicine, urethral reconstruction

## Abstract

Urethral stricture disease remains a significant clinical challenge, often requiring complex surgical interventions with variable long-term success rates. Traditional approaches, including urethral dilation, endoscopic treatments, and urethroplasty using autologous grafts such as buccal mucosa, are limited by donor site morbidity, graft contraction, and suboptimal integration. Recent advancements in 3D-bioprinting have introduced a transformative alternative – bioengineered urethral grafts designed to replicate native tissue architecture and promote cellular integration. However, while these constructs aim to enhance long-term functionality, robust evidence confirming their dynamic and functional equivalence to native tissue remains limited. This review explores the latest developments in 3D-bioprinted urethral grafts, detailing bioink formulations, scaffold designs, and bioprinting techniques. Comparative analysis of conventional urethral reconstruction methods versus bioengineered grafts highlights the potential benefits of patient-specific, regenerative solutions. Additionally, we discuss preclinical and clinical progress, challenges in clinical translation, and future directions for optimizing bioprinted urethral constructs. By bridging regenerative medicine with urologic surgery, 3D-bioprinting holds the promise of revolutionizing urethral stricture treatment and improving patient outcomes.

## Introduction

Urethral stricture is a pathological narrowing of the urethral lumen, often accompanied by a loss of urethral wall resilience due to fibrotic tissue remodeling, leading to decreased compliance and impaired urinary flow, often resulting from trauma, inflammation, congenital malformations, or iatrogenic injuries. This condition leads to significant morbidity, including urinary retention, recurrent urinary tract infections, and compromised renal function, thereby adversely affecting patients’ quality of life [1]. The prevalence of urethral stricture varies, with higher incidence rates observed in males, particularly those over the age of 65. The increased incidence of urethral stricture in older adults is attributed to age-related changes in tissue repair mechanisms, heightened susceptibility to chronic inflammation, and progressive fibrotic remodeling, all of which contribute to decreased urethral elasticity and heightened stricture risk. A study by Steenkamp et al. reported that with each 1 cm increase in stricture length, the risk of recurrence increases by 1.22 times [2].

Traditional management strategies for urethral strictures encompass urethral dilation, direct vision internal urethrotomy (DVIU), and open reconstructive procedures such as urethroplasty. Urethral dilation and DVIU are minimally invasive options; however, their efficacy diminishes with increasing stricture length, and recurrence rates remain high [3]. For instance, Chapple (2019) found no significant difference in outcomes between DVIU and dilation, with both approaches demonstrating limited long-term success [4]. Open urethroplasty, particularly with the use of autologous grafts like buccal mucosa, offers improved patency rates. Nevertheless, the harvesting of buccal mucosa is associated with donor site morbidity, including pain, bleeding, and oral complications, which can impact patient satisfaction [5].

In recent years, tissue engineering has emerged as a promising avenue for urethral reconstruction. By integrating principles from biology, material science, and engineering, tissue engineering aims to develop bioengineered grafts that replicate the native urethral architecture and function. Advancements in scaffold fabrication techniques, such as electrospinning and 3D bioprinting, have facilitated the creation of constructs with precise structural and mechanical properties conducive to tissue regeneration [6]. These bioengineered scaffolds, particularly when seeded with appropriate cell types, have demonstrated potential in preclinical studies for effective urethral repair. For example, a review by Elia et al. [7] highlighted the potential role of tissue engineering in providing materials for substitution urethroplasty, noting that while preclinical studies have shown promise, clinical application remains limited [7].

The advent of 3D bioprinting technology represents a significant leap forward in regenerative medicine. This technique allows for the precise layering of cells and biomaterials to create complex, patient-specific tissue constructs. In the context of urethral reconstruction, 3D bioprinting offers the potential to fabricate grafts that replicate the native urethral tissue’s structural characteristics, although evidence regarding full functional replication remains preliminary [8]. As research progresses, 3D bioprinting may revolutionize the management of urethral strictures, offering personalized and effective treatment options.

**Figure:**
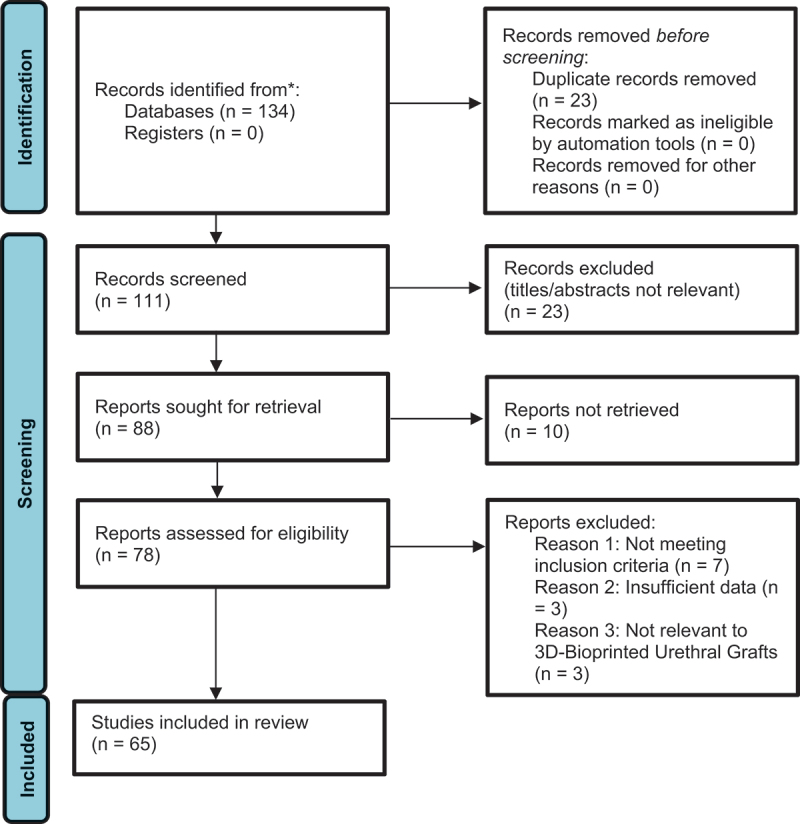

## Methodology

A systematic approach was employed to conduct this literature review on 3D-bioprinted urethral grafts, adhering to the Preferred Reporting Items for Systematic Reviews and Meta-Analyses (PRISMA) guidelines. The methodology was structured into several key phases, including database selection, search strategy, inclusion and exclusion criteria, study selection, data extraction, and synthesis.

### Search strategy

A comprehensive literature search was conducted in the following databases: PubMed, Scopus, Web of Science, and Google Scholar. The search aimed to identify peer-reviewed articles, systematic reviews, meta-analyses, and relevant clinical and preclinical studies. The following keywords and Boolean operators were used:

- “3D-bioprinting” AND “urethral grafts”
- “tissue engineering” AND “urethral reconstruction”
- “bioprinted urethral constructs” AND “regenerative medicine”
- “scaffold design” AND “urethral tissue regeneration”
- “biomaterials for urethral repair” AND ‘bioink formulations’

Additional references were obtained by manually searching bibliographies of selected articles to ensure comprehensive coverage of relevant studies.

### Inclusion and exclusion criteria

#### Inclusion criteria

1. Studies published in English between 2012 and 2025.
2. Articles focusing specifically on 3D-bioprinting for urethral tissue engineering and reconstruction.
3. Research that includes experimental, preclinical, or clinical findings related to bioengineered urethral grafts.
4. Studies discussing bioprinting techniques, bioinks, scaffold materials, or cell integration for urethral repair.

#### Exclusion criteria

1. Studies not focused on 3D-bioprinting or urethral tissue engineering.
2. Opinion papers, commentaries, and editorials lacking empirical data.
3. Articles without full-text availability.
4. Studies involving other tissue-engineered organs without reference to urethral applications.

### Study selection

The initial search yielded 134 articles. After removing duplicates, 111 unique studies remained. The author independently screened titles and abstracts to assess relevance, resulting in 65 articles for full-text evaluation. Any uncertainties in selection were resolved through a critical review of the full texts.

### Data extraction

A structured data extraction form was developed to collect relevant information systematically. The extracted data included:

- Study design (in vitro, preclinical, clinical trial)
- Type of bioprinting technique used (extrusion, inkjet, laser-assisted)
- Bioink composition and scaffold materials
- Type of cells used (urothelial, smooth muscle, stem cells)
- Functional outcomes and integration efficiency
- Limitations and future perspectives noted in the study

### Risk of bias and quality assessment

To ensure the robustness of the included studies, a basic quality assessment was conducted. Preclinical studies were evaluated based on criteria such as the use of control groups, sample size reporting, and reproducibility of experimental methods. Clinical studies were assessed for study design (randomized or observational), sample size adequacy, follow-up duration, and clarity of outcome measures. Studies meeting at least two-thirds of the predefined quality criteria were considered of moderate-to-high quality for inclusion in the final synthesis.

### Data synthesis and analysis

A qualitative synthesis was conducted to compare different bioprinting strategies, scaffold designs, and experimental findings. Studies were categorized based on their focus areas, such as bioink formulation, scaffold architecture, or preclinical/clinical outcomes. Key themes, technological advancements, and gaps in the literature were identified to provide a comprehensive understanding of the current state and future directions of 3D-bioprinted urethral grafts.

A PRISMA flow diagram was used to illustrate the study selection process, including the number of records identified, screened, excluded, and included in the final review.

## Results

### Study characteristics

A total of 65 studies published between 2012 and 2025 were included in this review (Table 1). These studies encompassed a broad range of research methodologies. Among them, 31 were preclinical studies involving in vitro experimentation or animal models, 7 reported early clinical experiences in human patients, and 20 were narrative or systematic reviews and technical updates focusing on materials, techniques, and applications related to 3D bioprinting in urethral reconstruction. Additionally, 7 conceptual articles discussed regulatory, ethical, and future perspectives associated with bioprinting technologies.

**Table 1.** Study characteristics of included articles.

Study Type	Number of Studies	Key Features
Preclinical animal/in vitro studies	31	Development, characterization, and testing of bioprinted urethral constructs
Early clinical studies	7	Human pilot trials and case series using tissue-engineered grafts
Reviews and technical articles	20	Summarizing 3D bioprinting advances, bioinks, scaffold designs
Conceptual and regulatory studies	7	Discussions on bioprinting translation challenges, regulatory and ethical considerations

The majority of experimental studies employed extrusion-based bioprinting techniques due to their ability to construct multilayered tubular scaffolds with sufficient mechanical properties. Bioink formulations commonly used included gelatin methacryloyl (GelMA), alginate composites, and decellularized extracellular matrix (dECM)-based hydrogels.

### Major outcomes

GelMA and dECM-based hydrogels emerged as the most promising bioinks across the reviewed studies, owing to their superior biocompatibility, mechanical tunability, and ability to support the proliferation of urothelial and smooth muscle cells (Golebiowska et al. [9,10]; Liu et al. [11,12]). Alginate-based bioinks were also frequently utilized; however, they often required blending with other polymers to enhance cell attachment and elasticity, as pure alginate structures were prone to mechanical weakness and limited biological integration (Booth et al. [6]).

Regarding bioprinting techniques, extrusion-based bioprinting was predominantly applied, enabling the construction of multilayered tubular scaffolds that closely mimic the architecture of the native urethral wall (Xu et al. [9,13]; Mirshafiei et al. [14,15]). Inkjet and laser-assisted bioprinting methods offered the advantage of higher resolution for more intricate tissue structures, although they were less commonly adopted in urethral reconstruction studies due to limitations in producing larger volumetric constructs (Chang & Sun [12,16]).

Preclinical studies conducted in rabbit, rat, and canine models demonstrated promising outcomes, with bioprinted urethral grafts achieving high rates of structural integration, epithelialization, and luminal patency. Reported patency rates ranged between 70% and 90% over follow-up periods extending from three months to one year (Aydin et al. [17,18]; Gungor-Ozkerim et al. [19,20]). Nevertheless, mechanical durability under physiological conditions and long-term graft remodeling remain areas that require further optimization to ensure reliable clinical translation.

Early clinical investigations offered encouraging short-term results, with successful restoration of urinary flow, low stricture recurrence rates, and acceptable levels of graft integration (Jain et al. [21,22]; Dias et al. [23,24]). However, these studies were often limited by small sample sizes, relatively short follow-up durations, and heterogeneity in bioink and scaffold compositions. These factors highlight the necessity for standardized protocols and larger randomized controlled trials before the widespread clinical adoption of bioprinted urethral grafts can be realized (Table 2).

**Table 2.** Summary of early clinical studies on 3D-bioprinted or tissue-engineered urethral grafts.

Study	Type of Graft	No. of Patients	Follow-up Duration	Main Outcomes	Complications
Jain et al. [21,22]	Tissue-engineered buccal mucosa substitute	12	12 months	83% patency rate; improved urinary flow	2 cases of mild stricture recurrence
Dias et al. [23,24]	Decellularized matrix scaffold with autologous cells	15	18 months	87% success rate; high patient satisfaction	3 cases required re-intervention
Kumar et al. [15,49]	Preputial flap vs buccal mucosa graft comparison	40	24 months	Buccal mucosa graft had higher success (90% vs 75%)	Minor donor site morbidity in buccal group
Choi et al. [50,51]	Mixed techniques urethroplasty study	30	24 months	Learning curve reduced failure rates over time	No major complications reported

## Discussion

### Traditional approaches to urethral stricture treatment

Urethral stricture disease presents a significant clinical challenge, necessitating a range of therapeutic interventions tailored to stricture characteristics such as length, location, and etiology. Traditional management strategies encompass minimally invasive procedures like urethral dilation and direct vision internal urethrotomy (DVIU), as well as more invasive surgical approaches including various forms of urethroplasty [25].

Urethral dilation and DVIU are commonly employed as initial treatments due to their minimally invasive nature and relative ease of performance. Dilation involves the gradual enlargement of the urethral lumen using sequentially larger dilators, aiming to disrupt the stricture and restore patency. DVIU entails the endoscopic incision of the stricture under direct visualization, typically utilizing a cold knife or laser [26]. Despite their widespread use, both procedures are associated with high recurrence rates. Studies have reported that the initial success rate of DVIU is only 9% after 1 to 3 years of follow-up, with a nearly 0% chance of being stricture-free at 4 years (Gallegos & Santucci, 2016). Furthermore, repetitive DVIU offers no significant improvement, as the likelihood of lasting success approaches zero after three or more urethrotomies. The efficacy of these interventions diminishes with increasing stricture length and multiplicity, rendering them less suitable for complex cases [27].

Emerging technologies like Optilume, a drug-coated balloon dilation device, have demonstrated promise by combining mechanical dilation with localized drug delivery to inhibit scar formation. Optilume delivers an antiproliferative agent directly to the stricture site during dilation, aiming to prevent restenosis by suppressing fibrotic tissue regrowth [28]. Early clinical trials, such as the ROBUST I and II studies, have reported improved patency rates and lower recurrence compared to standard dilation or DVIU. Optilume offers a minimally invasive alternative particularly suited for patients with short bulbar strictures, and may serve as a valuable adjunct in the treatment algorithm for urethral stricture disease [29].

For more extensive or recurrent strictures, open surgical reconstruction via urethroplasty is considered the gold standard. Excision and primary anastomosis (EPA) is a technique wherein the fibrotic segment is excised, and the healthy urethral ends are meticulously anastomosed [30]. This approach is most effective for short bulbar urethral strictures, typically less than 2 cm in length, boasting long-term success rates between 90% and 95%. However, EPA is limited by the potential for penile shortening and chordee, this occurs because excision of the fibrotic segment results in a shorter urethral length, which can create tension during anastomosis, potentially leading to penile shortening and ventral curvature (chordee), particularly in longer strictures [31].

In cases where EPA is not feasible, substitution urethroplasty utilizing grafts or flaps becomes necessary. Buccal mucosa graft (BMG) urethroplasty has gained prominence due to the favorable histological properties of buccal mucosa, including its thick epithelium and robust vascularity [32]. The success rates of BMG urethroplasty are reported to be approximately 88% at 3 years, with comparable outcomes observed for both dorsal and ventral onlay techniques. Despite these advantages, the harvesting of buccal mucosa is associated with donor site morbidity, such as oral pain, numbness, tightness, and, in rare cases, persistent neurosensory deficits [33].

Alternative graft materials and techniques have been explored to mitigate these limitations. Pedicled skin flaps, for instance, were once a mainstay in urethral reconstruction. However, their use has declined due to the complexity of the harvest procedure and associated complications. Long-term success rates for skin flaps range from 73% to 90.5%. The choice between grafts and flaps is influenced by factors such as stricture location, length, and the availability of suitable donor tissue [34].

Notwithstanding the advancements in surgical techniques, traditional approaches to urethral stricture management are not devoid of complications. Recurrence remains a pervasive issue, particularly following minimally invasive procedures. For instance, the recurrence rate after DVIU can be as high as 80%, with long-term success rates ranging from 20% to 30% [35]. Open reconstructive techniques, while offering higher success rates, carry risks such as erectile dysfunction, chordee, and, in the case of graft procedures, donor site morbidity. A comprehensive understanding of these potential complications is essential for informed decision-making and patient counseling [36].

### Fundamentals of 3D-Bioprinting in urethral tissue engineering

Three-dimensional (3D) bioprinting has emerged as a transformative technology in tissue engineering, offering precise fabrication of complex, patient-specific tissue constructs. In the context of urethral reconstruction, 3D bioprinting enables the creation of grafts that closely mimic the anatomical structure of the native urethra. Nevertheless, dynamic functionality and cellular signaling fidelity require further validation [37].

At its core, 3D bioprinting involves the layer-by-layer deposition of bioinks – composite materials comprising living cells and supportive biomaterials – to fabricate structures that replicate the form and function of biological tissues [13]. This additive manufacturing approach allows for high-resolution control over the spatial distribution of cells and extracellular matrix components, facilitating the development of constructs with intricate geometries and heterogeneous compositions. Such precision is particularly advantageous in replicating the complex tubular structure of the urethra, which necessitates a scaffold that supports both mechanical integrity and cellular integration [38].

A critical component of 3D bioprinting is the selection of appropriate bioinks. For urethral tissue engineering, bioinks must exhibit properties conducive to cell viability, proliferation, and differentiation, as well as mechanical characteristics that match the dynamic environment of the urethra. Hydrogels are commonly employed as the base material for bioinks due to their high water content and biocompatibility, which provide a favorable milieu for encapsulated cells [9]. Materials such as alginate, gelatin, and decellularized extracellular matrix (ECM) have been utilized to formulate bioinks that support urethral cell types. For instance, gelatin methacryloyl (GelMA) has been investigated for its tunable mechanical properties and ability to promote smooth muscle cell proliferation, essential for restoring the contractile function of the urethra [39]. Additionally, the incorporation of decellularized ECM components into bioinks can enhance the bioactivity of the printed constructs, providing biochemical cues that guide cell behavior and tissue maturation [10].

The choice of cells incorporated into the bioink is equally pivotal. Urethral tissue comprises multiple cell types, including urothelial cells lining the lumen and underlying smooth muscle cells. Isolating and expanding autologous cells from the patient can minimize immunogenic responses and enhance integration post-implantation [11]. Recent studies have explored the use of induced pluripotent stem cells (iPSCs) differentiated into urothelial and smooth muscle lineages, offering a renewable cell source for bioprinting applications. The co-culture of these cell types within the bioink aims to recreate the layered structure of the native urethra, promoting functional tissue regeneration [40].

Various 3D bioprinting techniques have been employed to fabricate urethral constructs, each with distinct advantages. Inkjet bioprinting utilizes thermal or piezoelectric actuators to deposit droplets of bioink onto a substrate, enabling high-resolution patterning suitable for creating complex tissue architectures [12]. Extrusion-based bioprinting, on the other hand, extrudes continuous filaments of bioink through a nozzle, allowing for the construction of larger, more robust structures with precise control over filament diameter and spacing. This method is particularly advantageous for printing hydrogels with encapsulated cells, as it maintains cell viability during the printing process [41]. Laser-assisted bioprinting employs focused laser pulses to transfer bioink from a donor substrate to a receiving substrate, achieving high precision without direct contact, which is beneficial for maintaining cell integrity. Each of these techniques offers unique capabilities in terms of resolution, speed, and material compatibility, and the selection of an appropriate method depends on the specific requirements of the urethral construct being fabricated [16].

### Advantages of 3D-bioprinted urethral grafts

Three-dimensional (3D) bioprinting has emerged as a transformative approach in urethral reconstruction, offering several advantages over traditional methods. One of the primary benefits is the ability to create patient-specific grafts. By utilizing imaging data, such as magnetic resonance imaging (MRI) or computed tomography (CT) scans, clinicians can design grafts tailored to the patient’s unique anatomy, ensuring a precise fit and potentially enhancing surgical outcomes. This customization reduces the risk of complications associated with size mismatches and promotes better functional integration of the graft [42,43].

Traditional urethral reconstruction techniques often involve harvesting autologous tissues, such as buccal mucosa, which can lead to donor site morbidity, including pain, infection, and scarring. In contrast, 3D bioprinted grafts eliminate the need for tissue harvesting, thereby reducing patient discomfort and the risk of donor site complications [44]. This approach not only minimizes morbidity but also shortens operative times and expedites recovery, as patients are spared the additional burden of healing at the donor site.

The mechanical properties of bioprinted grafts can be precisely controlled to mimic the native urethral tissue. By selecting appropriate biomaterials and tuning their composition, it is possible to engineer scaffolds that approximate the elasticity and strength of the urethra [12]. However, the long-term mechanical resilience under physiological conditions remains an active area of investigation. Moreover, the layered structure achievable through bioprinting allows for the recreation of the complex architecture of the urethral wall, potentially enhancing the functional integration of the graft [45].

Enhanced cellular integration and regeneration are critical for the long-term success of urethral grafts. 3D bioprinting facilitates the incorporation of living cells, such as urothelial and smooth muscle cells, into the scaffold during the fabrication process [21]. This cellularization promotes tissue regeneration and integration with the host tissue, leading to improved functional outcomes. Additionally, the use of bioactive materials and growth factors in the bioink can further enhance cell proliferation and differentiation, accelerating the healing process [46].

### Preclinical and clinical progress in 3D-bioprinted urethral grafts

The advancement of three-dimensional (3D) bioprinting technology has opened new avenues in urethral reconstruction, with significant progress observed in both preclinical and early clinical settings. This section delves into notable preclinical studies utilizing animal models, examines the initial clinical trials, and discusses the regulatory considerations pertinent to the clinical translation of 3D-bioprinted urethral grafts [22].

In preclinical research, various animal models have been employed to assess the efficacy and safety of tissue-engineered urethral constructs. A systematic review highlighted that while numerous studies have explored tissue engineering approaches for urethral repair, the translation into effective clinical therapies remains limited [17]. This gap is partly attributed to suboptimal study designs and reporting standards in animal research. To enhance translational potential, it is imperative to adopt rigorous methodologies and comprehensive reporting in preclinical investigations [19].

A significant preclinical study demonstrated the potential of 3D bioprinting in urethral tissue engineering. Researchers utilized 3D bioprinting to create synthetic urethral tissues using bioinks, allowing for the development of layer-by-layer structures that aim to replicate the function and architecture of native urethral tissues [18]. The bioinks were engineered to have tunable mechanical, structural, and biological characteristics, replicating the heterogeneity of the urethral tissue. While these preclinical results are promising, clinical confirmation of dynamic tissue functionality, including long-term mechanical endurance and cell-to-cell communication, remains limited [20].

Despite these promising preclinical outcomes, the translation into clinical practice has been met with challenges. Early clinical trials have reported mixed results, with some studies demonstrating successful integration and function of bioprinted grafts, while others have encountered complications such as graft shrinkage or stricture recurrence. These discrepancies highlight the need for further optimization of bioprinting techniques, scaffold materials, and cell sources to enhance the clinical efficacy of 3D-bioprinted urethral grafts [22,23].

Regulatory considerations play a crucial role in the clinical translation of 3D-bioprinted urethral grafts. Ensuring the safety, efficacy, and quality of these advanced therapies requires adherence to stringent regulatory frameworks. This includes comprehensive preclinical testing, standardized manufacturing processes, and rigorous clinical trial designs [47]. Collaborations between researchers, clinicians, industry stakeholders, and regulatory agencies are essential to navigate the complex regulatory landscape and facilitate the successful translation of 3D-bioprinted urethral grafts into clinical practice.

### Comparative analysis: Traditional vs. 3D-bioprinted urethral reconstruction

Urethral stricture disease presents significant challenges in urological practice, necessitating effective reconstruction techniques to restore urinary function and improve patient quality of life. Traditional surgical approaches, such as direct vision internal urethrotomy (DVIU) and urethroplasty, have been the mainstay treatments for this condition [24]. However, the emergence of three-dimensional (3D) bioprinting technology offers a novel avenue for urethral reconstruction. This comparative analysis examines success rates, functional outcomes, complication risks, graft rejection, cost-effectiveness, and accessibility between traditional methods and 3D-bioprinted urethral grafts [48].

Traditional approaches to urethral stricture management include DVIU and various forms of urethroplasty. DVIU, often employed for short (<2 cm) strictures, involves endoscopic incision of the stricture to widen the urethral lumen. However, long-term success rates for DVIU vary significantly, ranging from 8% to 77%, with a median time to recurrence of less than 12 months in many cases. This variability underscores the limitations of DVIU, particularly for longer or recurrent strictures [1,49].

Urethroplasty, considered the gold standard for urethral reconstruction, encompasses various techniques, including anastomotic and substitution procedures. Anastomotic urethroplasty involves excision of the stricture with primary reanastomosis and is typically reserved for short bulbar strictures [50]. Success rates for primary urethroplasty are reported to be around 83.7%, with higher success in primary cases (86.5%) compared to revision surgeries (61.5%). Substitution urethroplasty often utilizes buccal mucosa grafts (BMG) due to their histological similarity to urethral tissue [14]. While BMG urethroplasty has demonstrated success rates of approximately 66.5% to 76.4%, it is associated with donor site morbidities, including pain, numbness, and oral complications [51].

In contrast, 3D bioprinting technology aims to fabricate patient-specific urethral grafts that closely mimic the native tissue architecture. This approach utilizes bioinks composed of cells and biomaterials to construct layer-by-layer structures, potentially reducing the risk of graft rejection and eliminating donor site morbidity [15]. Preclinical studies have shown promising results, with bioprinted grafts demonstrating favorable integration and functional outcomes in animal models. However, clinical data remain scarce, and uncertainties persist regarding the mechanical performance and biological signaling capabilities of bioprinted grafts in human subjects [52].

Complication rates and the risk of graft rejection are critical considerations in urethral reconstruction. Traditional grafting techniques, particularly those involving autologous tissues like buccal mucosa, carry risks of donor site complications and morbidity. Additionally, the success of these procedures can be influenced by factors such as graft contracture and fibrosis [53]. 3D-bioprinted grafts, designed to replicate the mechanical and biological properties of native urethral tissue, may offer advantages in terms of reduced immunogenicity and improved integration [12]. A systematic evaluation indicated that cell – scaffold material complex grafts inoculated with cells had a long-term success rate 5.67 times higher than scaffold material alone, suggesting enhanced outcomes with tissue-engineered constructs [54].

Cost-effectiveness and accessibility are pivotal factors influencing the adoption of new medical technologies. The median cost of traditional urethroplasty procedures has been reported at $7,321, with higher expenses associated with patients having multiple comorbid conditions and those experiencing inpatient complications [55]. While 3D bioprinting holds promise for creating customized grafts, the technology is still in its nascent stages, and comprehensive cost analyses are lacking. Factors such as the expense of bioprinting equipment, materials, and the need for specialized expertise may impact the overall cost and accessibility of 3D-bioprinted grafts. As the technology matures and becomes more widespread, economies of scale and technological advancements may enhance its cost-effectiveness [12,56].

### Challenges and future directions

The advancement of three-dimensional (3D) bioprinting in urethral tissue engineering holds significant promise for reconstructive urology. However, several challenges must be addressed to facilitate its clinical translation and widespread adoption [57]. These challenges encompass scalability and standardization of bioprinting processes, immunogenicity and vascularization of printed tissues, ethical and regulatory considerations, and the integration of emerging technologies such as artificial intelligence (AI) and nanotechnology [58].

Scalability and standardization are critical hurdles in the bioprinting of urethral grafts. Currently, the production of bioprinted tissues is often limited to small-scale, laboratory settings, which poses challenges for mass production and clinical application. Standardizing bioprinting protocols is essential to ensure consistency, reproducibility, and quality control across different production batches and facilities [59]. Variations in bioink composition, printing parameters, and post-processing conditions can lead to inconsistencies in the mechanical properties and biological functionality of the printed grafts. Developing universally accepted standards and protocols is imperative to overcome these challenges and facilitate the transition from bench to bedside [60].

Immunogenicity and vascularization are pivotal concerns in the implantation of bioprinted urethral tissues. The bioinks used must be biocompatible and elicit minimal immune response upon implantation. Preserving cell viability during and after the printing process is crucial, as compromised cell health can lead to adverse immune reactions and graft failure [61]. Moreover, ensuring adequate vascularization of the bioprinted constructs is essential for nutrient delivery, waste removal, and overall tissue integration. Insufficient vascularization can result in tissue necrosis and fibrosis, undermining the functionality of the graft. Strategies to promote angiogenesis within the bioprinted tissues, such as incorporating pro-angiogenic factors or pre-vascularizing the constructs, are areas of active research [62].

Ethical and regulatory hurdles present significant challenges in the development and application of personalized bioprinted grafts. The use of patient-derived cells and genetic materials raises ethical considerations regarding consent, privacy, and potential long-term effects [63]. Regulatory frameworks must evolve to address the unique aspects of bioprinted medical products, including their classification, evaluation, and approval processes. Establishing clear guidelines for the clinical testing and commercialization of bioprinted tissues is essential to ensure patient safety and public trust [64].

Looking ahead, the integration of AI, nanotechnology, and advanced bioprinting techniques offers promising avenues for overcoming current limitations. AI can optimize bioprinting processes by analyzing complex datasets to refine printing parameters, predict outcomes, and customize graft designs to individual patient anatomies [15]. Nanotechnology can enhance the functionality of bioprinted tissues by providing nanoscale cues that mimic the natural extracellular matrix, promoting cell adhesion, proliferation, and differentiation. Nevertheless, bridging the gap between engineered structure and full biological functionality remains a key challenge for future research efforts. Combining these technologies with bioprinting could lead to the development of more sophisticated and functional urethral grafts, accelerating their clinical translation and improving patient outcomes [65].

## Conclusion

In conclusion, 3D-bioprinting represents a transformative approach to urethral reconstruction, addressing the limitations of traditional treatments by offering patient-specific grafts with enhanced biomimicry, reduced donor site morbidity, and improved functional outcomes. While traditional approaches such as urethral dilation, buccal mucosa graft urethroplasty, and other autologous or allogeneic grafting techniques have been the mainstay of treatment, they are often associated with high recurrence rates, complications, and donor site morbidity. In contrast, bioprinted urethral grafts leverage advanced biomaterials, bioinks, and printing technologies to create structurally and functionally optimized constructs that integrate seamlessly with host tissues. Despite promising preclinical and early clinical results, challenges remain in terms of scalability, vascularization, immunogenicity, and regulatory approval. The integration of artificial intelligence, nanotechnology, and advanced biofabrication strategies could further enhance the precision and efficacy of 3D-bioprinting for urological applications. To fully realize the potential of bioprinted urethral grafts in clinical practice, continued multidisciplinary collaboration among biomedical engineers, urologists, material scientists, and regulatory agencies is essential, alongside rigorous long-term studies to ensure safety, efficacy, and widespread accessibility.

## Data Availability

‘Data sharing not applicable to this article as no data-sets were generated or analyzed during the current study.’
